# The Influence of Grandparents on Children’s Dietary Health: A Narrative Review

**DOI:** 10.1007/s13668-023-00483-y

**Published:** 2023-06-17

**Authors:** Michelle I. Jongenelis, Timothy Budden

**Affiliations:** 1 Melbourne Centre for Behaviour Change, Melbourne School of Psychological Sciences, The University of Melbourne, Parkville, 3010 Australia; 2 School of Human Sciences (Exercise and Sport Science), The University of Western Australia, Perth, 6008 Australia; 3 Telethon Kids Institute, Perth, 6008 Australia

**Keywords:** Grandparents, Grandchildren, Caregiving, Dietary health, Nutrition

## Abstract

***Purpose of Review*:**

To examine and synthesise recent evidence on the role of grandparents in shaping children's dietary health.

***Recent Findings*:**

The influence of grandparents on children’s dietary health was evident across studies. Grandparents frequently provide their grandchildren with meals and snacks, and engage in many of the same feeding practices used by parents. Although grandparents report providing their grandchildren with healthy foods, the provision of treat foods high in sugar or fat was a common finding. This provision led to family conflict, with the indulgent behaviours of grandparents seen by parents as a barrier to healthy eating.

***Summary*:**

Grandparents are exerting significant influence on child dietary health. Efforts are needed to ensure these care providers are considered key stakeholders in the promotion of healthy eating and are targeted in policies and programs addressing children’s diets. Research that determines how to best support grandparents to foster healthy behaviours in children is critical.

**Supplementary Information:**

The online version contains supplementary material available at 10.1007/s13668-023-00483-y.

## Introduction

Poor nutrition has been highlighted as a key modifiable factor that plays a crucial role in the development and maintenance of multiple non-communicable diseases [1]. The promotion of a diet characterised by (i) adequate consumption of fruit, vegetables, and wholegrains and (ii) infrequent consumption of energy-dense nutrient-poor foods and beverages is thus considered important to health outcomes, with such a diet reducing the risk of all-cause mortality and diseases such as type 2 diabetes and cancer [2–6].

Given that dietary habits established early in life track into adulthood [7], the promotion of a healthy diet in childhood to reduce the risk of diet-related chronic disease at all stages of the life course has been identified as a global health priority [8]. A range of individual, familial, social, and environmental factors shape children’s eating behaviours [9]. The role of parents is considered particularly important. Parents influence their children’s diet directly as gatekeepers of the eating environment and indirectly through their role as nutrition educators and modellers of food choice [10–12]. Although parents remain critical, recent decades have seen societal factors such as increased maternal participation in the workforce and the reduced affordability, availability, and flexibility of formal childcare arrangements contribute to worldwide increases in grandparents’ involvement as secondary care providers to their grandchildren [13–17]. Accordingly, it has been suggested that grandparents be considered important stakeholders in the promotion of healthy eating among children [18•]. The purpose of this review was to examine and synthesise recent work exploring the role of grandparents in shaping children's dietary health and eating behaviours.

## Method

### Search Strategy and Selection Criteria

A comprehensive search of the following databases was conducted for original research articles published from 1^st^ January 2013 to the 31^st^ December 2022: Google Scholar, EBSCO, Medline, PubMed, ProQuest, Science Direct, SCOPUS, and Web of Science. The search terms were (grandparents OR grandcarers OR grandmothers OR grandfathers) AND (grandchildren OR grandkids) AND (diet OR nutrition OR feeding). To be included in this review, studies must have been available in full text and published in English. Only studies exploring the role of non-custodial, non-residing grandparents were eligible for inclusion; studies involving custodial or co-residing grandparents were excluded. A separate search was conducted for meta-analyses and systematic reviews. These are not included in this narrative review, but a list of relevant reviews is presented in the online supplementary material.

A total of 2,167 studies was identified. After screening these for relevance and eligibility, 25 studies remained.

## Findings

A summary of each of the studies reviewed is presented in Table 1. The influence of grandparents on children’s dietary health was evident across all studies. The sections below outline the findings according to specific areas of influence.

**Table 1:** Summary of studies reviewed

Reference	Location	Objective(s)	Methodology	Sample	Results^a^
Bektas et al. [31]	Netherlands	Explore the influence of grandmothers on the health-related practices of their grandchildren during the first 1000 days	Qualitative Focus groups (*n* = 3) and individual interviews with grandmothers and mothers	Turkish grandmothers (*n* = 29) and mothers (*n* = 16) living in the Netherlands with a (grand)child aged 0–4 years for whom they provide care at least 2 times a week	Influence of grandmothers on their grandchildren’s health-related practices was evident and substantial Mothers can perceive the guidance and pressure they receive from grandmothers as stressful Grandmothers and mothers tend to experience conflict related to differing feeding-related views and practices when grandmothers babysit Both parties find discussing these differences difficult, fearing family conflict
Casteñada-García et al. [27]	Canary Islands	Explore diet and physical activity in grandchildren by: 1. Obtaining a sociodemographic profile of grandparents interviewed; 2. Establishing what type of foods grandparents serve grandchildren, and whether these relate to the sociodemographic variables; and 3. Analysing the types of physical activity shared between grandparents and grandchildren and whether these are related to the sociodemographic variables	Quantitative Verbally administered survey of Spanish grandparents	Spanish grandparents (*n* = 114; 83% women) with grandchildren > 2 years old	Grandparents reported mostly serving grandchildren the types of foods more likely to favour overweight (e.g. cookies, processed juices), but there were no statistically significant differences with other foods grandparents served that were less likely to favour overweight (e.g. milk, oats)
Chambers et al. [23]	Scotland	Examine the care practices of grandparents in families living in areas of high deprivation and consider the extent to which grandparents could be at the centre of health-promoting initiatives for children	Qualitative Individual interviews with grandmothers and mothers	Grandmothers (*n* = 15) and mothers (*n* = 15) living in areas of high deprivation Grandparents were caring for grandchildren < 16 years at least once a month	Grandparents' care practices were described as either responsible or fun Some grandmothers provided an authoritative approach, engaging in practices perceived to enhance their grandchildren’s wellbeing in a way that aligned with recommendations around diet. Other grandmothers expressed pride that they provided grandchildren with home cooked meals. Some grandparents described their remit as extending to discretionary foods as well as meals. They reported limiting their grandchildren’s consumption of foods considered less healthy and developing rules to manage this consumption Some grandparents considered themselves treat providers and spoiled their grandchildren with high sugar or fat snacks or takeaway meals Tension between grandparents and parents regarding caregiving practices was reported
Criss et al. [25]	United States	Explore food perceptions among grandparents and understand the influence of these perceptions on food choice for the younger generations in their family	Qualitative Focus groups (*n* = 14) with grandparents	Black, Hispanic, and White grandparents (*n* = 58; 72% women)	Grandparents’ perceived influence on their children’s and grandchildren’s food choices was described through the themes of (i) proximity and power, (ii) healthy vs. unhealthy spoiling, (iii) cultural food tradition, and (iv) reciprocal exchange of knowledge Some grandparents reported that the grandparent role allowed them to spoil their grandchildren with sugary or fried foods because their parents were primarily responsible for food provision
Eli et al. [28]	United States	Elucidate parents’ and grandparents’ perspectives on young children’s feeding and physical activity and identify how they negotiate potential differences between these perspectives	Qualitative Individual interviews with parents and grandparents	Parents (*n* = 22) and grandparents (*n* = 27) from 16 families with children aged 3–5 years	Three themes related to feeding practices were identified: 1. Disagreements about feeding stem from parents’ and grandparents’ differing definitions of healthy eating 2. Differences between parents’ and grandparents’ feeding practices reflect differences in perceived caretaking roles 3. Parents and grandparents negotiate differences in feeding practices through grandparental compliance and parental compromise
Eli et al. [24]	United States	Examine mothers’ and maternal grandmothers’ attitudes, knowledge, and practices regarding pre-school aged children’s beverage consumption	Qualitative Individual interviews with mother-maternal grandmother dyads	Predominantly White/Caucasian mothers (*n* = 11) and grandmothers (*n* = 11) (maternal dyads) of a child aged 3–5 years. Grandmothers were actively involved in their grandchild’s life (i.e. spent time with their grandchild at least 2 × per month)	Three themes were identified: 1. Mothers and grandmothers agreed on the hierarchy of healthiness between and within beverages, though fruit juice occupies an ambivalent position 2. Mothers and grandmothers cited role modelling and the home environment as important in regulating pre-schoolers’ beverage intake 3. Mothers and grandmothers reported balancing between restricting sugary beverages and using these beverages as treats
Farrow [36]	United Kingdom	Explore: 1. Whether differences between parents and grandparents exist in terms of their feeding practices; and 2. Whether grandparents’ feeding practices are related to the number of hours that they spend caring for grandchildren	Quantitative Hard copy survey of parents and grandparents	Parents (*n* = 50; 98% female) and grandparents (*n* = 50; 78% female) of grandchildren aged 2–8 years Participants were derived from two distinct and unrelated groups (i.e. no family parent-grandparent dyads)	Encouraging balance was the most common feeding practice used by grandparents, followed by providing a healthy eating environment Compared to parents, grandparents reported using significantly more maladaptive feeding practices (e.g. using food to regulate emotions; restricting food) and significantly more positive practices (e.g. providing a healthy food environment) The more hours grandparents spent caring for children, the more their feeding practices resembled those broadly reported by parents
Glover et al. [26]	New Zealand	Explore Māori caregivers’ views of the relative importance of weight to health, and the facilitators and barriers to a healthy weight in children aged 6 months to 5 years	Qualitative Focus groups with caregivers (*n* = 5)	Caregivers (e.g. parents and grandparents; *n* = 37) of children aged 6 months to 5 years residing in lower socioeconomic status areas	Parents expressed frustration that grandparents fed children what the children wanted, as well as special treats
Hemar-Nicolas et al. [37]	France	Investigate the interweaving of the socialisation systems within which children learn eating practices	Qualitative Individual interviews with children	Children aged 7–10 years (*n* = 20; 55% girls)	Grandparents found to be important agents of food-related socialisation Grandparents held an important role teaching food consumption to their grandchildren Grandparents were also responsible for transmitting knowledge about nutrition
Jongenelis et al. [18•]	Australia	Assess the extent to which Australian grandparents are providing meals and snacks for their grandchildren, the types of foods and beverages being provided, and the determinants of provision	Quantitative Online survey of grandparents	Grandparents (*n* = 1076; 60% women) who provide regular care (i.e. ≥ 3 h per week) to a grandchild aged 3–14 years	Vast majority of grandparents (98%) provided at least 1 meal or snack Snack provision was the most common (82%), followed by lunch (57%), then dinner (48%) and breakfast (40%) One in five (18%) provided their grandchildren with breakfast, lunch, dinner, and snacks Fresh fruit; milk, cheese, or yoghurt; vegetables; grain and cereal foods; and meat and meat alternatives were most frequently provided Sugary drinks and legumes were provided least frequently
Jongenelis et al. [35••]	Australia	Examine the feeding practices of grandparents who report providing childcare to their grandchildren Develop and test a model linking the various practices of grandparent caregivers to the frequency with which their grandchildren consume healthy and unhealthy foods while in grandparental care Explore the sociodemographic predictors of engagement in feeding practices	Quantitative Online survey of grandparents	Grandparents (*n* = 1076; 60% women) who provide regular care (i.e. ≥ 3 h per week) to a grandchild aged 3–14 years	Grandparents reported using positive feeding practices (encouragement of balance and variety; provision of a healthy food environment; modelling of healthy eating; limit setting; provision of praise) more frequently than negative feeding practices (control over eating; pressure to eat; instrumental feeding; emotional feeding) Encouragement of balance and variety was the most frequently used positive feeding practice Control over eating was the most frequently used negative feeding practice Positive feeding practices were found to be more important correlates of diet quality than negative feeding practices. Providing a healthy food environment and limit setting were associated with favourable dietary behaviours
Jongenelis et al. [39]	Australia	Explore the issues encountered by grandparents when providing their grandchildren with healthy food and the strategies they use to overcome these barriers	Qualitative Focus groups (*n* = 10) with grandparents	Grandparents (*n* = 79; 58% women) who provide regular care (i.e. ≥ 3 h per week) to at least one grandchild aged 3–12 years	The most commonly perceived barriers to providing grandchildren with healthy foods were children’s food preferences, the promotion of unhealthy food consumption by grandchildren’s parents, advertising of unhealthy food, and peer pressure. Most commonly used strategies to increase healthy food consumption and minimise unhealthy food consumption were disguising fruit and vegetables, making healthy foods appealing, involving grandchildren in food preparation and cooking, and rewarding grandchildren for healthy food consumption
Kim et al. [40]	South Korea	Identify the barriers in home and school settings that hamper healthy eating in overweight and obese children in South Korea	Qualitative Focus groups (*n* = 4) with children (*n* = 2 focus groups) and parents (*n* = 2 focus groups)	Children (*n* = 15; 33% girls) and parents (*n* = 15; 93% women)	Participants were aware of the importance of home and school environments in shaping children’s eating habits Parents expressed concerns about the permissiveness of grandparents Parents highlighted inconsistencies between parents and grandparents in enforcing restrictions on unhealthy food for their overweight or obese children
Knight et al. [32]	United Kingdom	Explore the place of childhood memories and intergenerational relations in the transmission of family food practices	Qualitative Interviews with parents and children	Families (*n* = 48) with children aged 1.5–10 years	Most mothers were happy with the meals children ate at their grandparents’ home. Some children and their mothers reported having greater variety of food because of the grandparents’ direct involvement in providing them with meals Providing food to grandchildren raised issues about what is acceptable and healthy for children to eat, with varying values and norms held by parents and grandparents A common complaint made by mothers about grandparents caring for their children was that they indulged them too much with unhealthy ‘treats’. Mothers wanted to exercise their own influence over what their children ate at the grandparents’ home but were also reliant on grandparents to provide care and did not want to disturb the grandparent-grandchild relationship
Lidgate et al. [41•]	United Kingdom	Explore parents’ and informal caregivers’: 1. Experiences in receiving or giving informal care for children aged 0–5 years; 2. Perceived explanations of the relationship between informal childcare and childhood obesity; and 3. Preferred intervention ideas and delivery strategies for preventing obesity among those children under informal care	Qualitative Focus groups (*n* = 4) with parents (*n* = 2 focus groups) and informal caregivers (*n* = 2 focus groups)	Parents (*n* = 7) and informal caregivers (*n* = 7) of a child aged 0–5 years	Cross-generation (parent and grandparent) conflict was perceived to prevent the adoption of healthy practices In exchange for receiving care for their children, parents lost control over what their child ate. They did not want to affect their relationship with the informal caregiver and so felt unable to provide healthier suggestions A common view held by parents was that grandparent care was more lenient. Both parents and informal caregivers were aware that grandparents treated their grandchildren with sugary foods, such as sweets and chocolates
McArthur et al. [19]	United States	Measure snack-related practices, beliefs, and awareness of grandparents providing informal childcare	Quantitative Self-administered, paper-and-pencil questionnaire	Grandparents (*n* = 78; 95% women) who provided informal care for ≥ 1 pre-school or school-aged grandchildren for ≥ 1 h per week in the grandparents’ residence and offered at least one snack per childcare occasion	On average, grandparents offered grandchildren 2.75 snacks per caregiving occasion. Nearly one-fifth (18%) of grandparents offered ≥ 4 snacks per occasion The strongest reported influences on snack purchases were perceived healthiness of the products (54%) and grandchildren’s preferences (47%) In terms of grandparents’ beliefs about the healthiness of their snack offerings, 48% reported providing ‘some unhealthy and some healthy’ snacks, 44% reported providing ‘mostly healthy’ snacks, and 8% provided ‘mostly unhealthy’ snacks Almost three-quarters (72%) of grandparents believed the snacks they provided would have a ‘mostly good’ effect on their grandchildren’s long-term health Grandparents typically offered snacks when they were requested (64%) or when the grandparent was having a snack and wanted their grandchild to have one too (46%) When grandchildren were naughty, grandparents’ self-efficacy for offering healthy snacks was lowest. The most frequently reported barrier to offering healthy snacks was grandchildren’s dislike for the taste of healthy snacks (51%) and cost (45%)
Marr et al. [34••]	United Kingdom	Explore the similarities and differences between parent and grandparent dietary provision, feeding practices, and feeding styles to pre-school-aged children	Quantitative Online survey of parents and grandparents	Unrelated parents (*n* = 72; 100% women) and grandparents (*n* = 44; 85% women) of children aged 2–4 years. Grandparents were eligible to participate if they reported caring for their grandchild ≥ 1 day per week and provided ≥ 1 meal	Parents and grandparents were providing meals high in saturated fat and sodium. Fruit and vegetables were provided at levels below the recommended amount There were no significant differences between parents and grandparents on the nutritional content of breakfast, lunch, dinner, and snacks served There were no significant differences between parents and grandparents on the amount of fruit and vegetables served at mealtimes Fruit as part of a snack was provided by 66% of parents and 61% of grandparents. Vegetables as part of a snack were provided by 4% of parents and 11% of grandparents. Discretionary food items were provided by 64% of parents and 61% of grandparents An indulgent feeding style was the most common among grandparents (41%) followed by authoritative (23%) then uninvolved and authoritarian (both 18%). There was no significant difference in feeding style between grandparents and parents In terms of feeding practices, promoting balance and variety was the most frequently reported among both parents and grandparents. The least frequently reported was using food to regulate emotions. Grandparents were significantly more likely than parents to report creating a healthy environment. They were less likely than parents to report using food as a reward and promote balance and variety
Mena et al. [42]	United States	Explore 1. Precursors and contextual influences on parental feeding; and 2. Parental perceptions and knowledge of the childcare food environment	Qualitative Focus groups (*n* = 4) with parents	Hispanic mothers (*n* = 34) and grandmothers (*n* = 2) of children aged 2–5 years	Mothers reported that culture and bigenerational differences between primary caregivers and grandparents influenced what their children ate Mothers reported struggling with their parents when they fed their children non-traditional foods Mothers believed that grandparents’ indulgent behaviours undermined their efforts to provide a healthy eating environment and promote healthy eating habits A few mothers reported that not indulging children was taken very seriously by grandparents and was considered abusive because eating sweets should be part of being a child
Metbulut et al. [38]	Turkey	Evaluate and compare mothers’ and grandmothers’ feeding behaviours and the relationship between grandparents’ feeding behaviours and children’s (i) feeding problems and (ii) body mass index	Quantitative Questionnaire administered to mothers and grandmothers Children’s body mass index measured by the researchers	Mothers (*n* = 150) and grandmothers (*n* = 50) of children aged 2–5 years *N* = 200 children represented (51% boys)	Restriction of food for health and weight reasons was the most frequently used feeding practice reported by grandmothers, followed by modelling of eating behaviour and then allowing children to have control during mealtimes and over food choice. The least frequently used feeding practice was the use of food for emotion regulation Grandmothers were more likely than mothers to use food for emotion regulation. They were less likely than mothers to encourage energy, balance, and variety; monitor child food intake; restrict food for health and weight reasons; and teach about nutrition
Neuman et al. [33]	United States	Examine how parents and grandparents describe their provision of food to pre-school aged children	Qualitative Interviews with parents and grandparents	Parents (*n* = 22) and grandparents (*n* = 27) from 16 families. All participants were parents or grandparents of a child aged 3–5 years Grandparents spent time with grandchild ≥ 2 occasions per month	Grandmothers were involved in food provision Grandmothers were identified as role models for dietary intake
O’Donohoe et al. [22]	Denmark and New Zealand	Understand the food consumption practices involved in grandparent—grandchild identity bundles	Qualitative Individual and joint interviews with grandparents and grandchildren	Grandparents (*n* = 23) and grandchildren (*n* = 17) from 18 families Grandchildren were aged 6–28 years	Grandparents' and grandchildren’s time alone together was considered a time for treats, with treats becoming embedded in grandparent–grandchild regular routines and relationships Treats and snacks played a significant role in grandparents’ food practices, but many grandparents and grandchildren highlighted that spoiling and unhealthy food consumption were undertaken in moderation Grandparents reported encouraging consumption of fruit, vegetables, and “proper meals,” and limiting intake of unhealthy food. Grandchildren reported eating “proper meals” but also treat foods Food rituals, routines, and rules between grandparents and grandchildren differed from family time shared by children and parents. Grandparents took pleasure in subverting parental rules around healthy eating. In some cases, however, grandparents explicitly deferred to parental preferences and practices Food practices were described as fun and special. Almost all grandparents reported being keen to provide meals that their grandchildren like to eat and took pride in their grandchildren’s enjoyment of their “special” dishes Agreement between both generations that grandparents generally had more time for cooking meals from scratch, whereas dinner provided at home by busy parents was often functional Good behaviour was rewarded with treats
Pankhurst et al. [29••]	Australia	Explore the meaning and role of food treats among grandparents who provide regular informal care to young grandchildren	Qualitative Interviews and focus groups with grandparents	Grandparents (*n* = 12) caring for grandchild(ren) aged 1–5 years for ≥ 10 h per week	Grandparents discussed the use of treats (i) in behavioural or emotional situations, (ii) as an educational tool, and (iii) as an expression of love and care Most grandparents were opposed to using food treats for comfort, to ameliorate negative emotions, or to control outbursts such as tantrums. Instead, most viewed food treats as an appropriate reward for an accomplishment, to reinforce good behaviour, and to teach manners Most grandparents believed that it was important for children to be exposed to discretionary foods so that they could learn to balance and moderate their intake and apply self-control Most grandparents felt that providing food treats to their grandchildren while in adult settings was a way to help them feel included and teach them social etiquette Many grandparents discussed the demands on modern parents and feared that grandchildren were ‘missing out’ on a variety of childhood experiences due to parents’ lack of time and money. They sought to compensate by providing grandchildren with core foods and treats that parents could not provide Grandparents did not claim the same role or responsibility as parents but some felt an increased sense of responsibility for their grandchildren Most grandparents discussed the importance of modelling healthy behaviours and being mindful of their own diet when grandchildren were present Grandparents restricted food treats when a main meal was imminent Grandparents felt that as their family feeding practices had been passed down through generations, food-related rules and beliefs were relatively consistent between care environments resulting in a low level of conflict between grandparent and parent. The beliefs and practices of grandparents and parents did differ occasionally, however. The manner in which grandparents approached these differences varied. Some grandparents felt parents' rules were overly strict and sought to counterbalance with a softer, more lenient approach. Others respected parents’ rules even when they disagreed with them All grandparents mentioned the importance of consistency Grandparents seemed to enjoy being viewed by their grandchildren as indulgent but did not want to be manipulated or taken for granted
Rhodes et al. [20]	Australia	Explore how decision-making and behaviour focused on food choices operates within the broader family context	Qualitative Family interviews with children, parents, and grandparents Family must have at least one child aged between 7 and 18 years	*N* = 27 three generation families: *n* = 11 Anglo-Australian families *n* = 8 Chinese-Australian families *n* = 8 Italian-Australian families *N* = 114 participants: *n* = 35 children (60% girls) *n* = 43 parents (63% female) *n* = 36 grandparents (67% female)	Grandparents caring for children before and after school had requests made of them to prepare family meals according to the children’s likes and dislikes Grandmothers who regularly prepared the family’s evening meal appeared to be influenced by children who indicated a dislike for their grandparents' diet Grandparents, particularly in Chinese and Italian families, exerted some influence on family food consumption through the use of traditional recipes Grandparents were not seen as being responsible for shaping the family food environment of their grandchildren Grandmothers appeared less concerned than mothers for the dietary health of the extended family, particularly grandchildren, and expressed being more lenient with treat foods
Rogers et al. [30••]	Australia	Gain insight into the perspectives of grandparents as informal carers of grandchildren with regard to their role in feeding their grandchildren aged 1–5 years	Qualitative Interviews with grandparents	*N* = 11 grandparents (82% female) who had at least 1 grandchild aged 1–5 years for whom they provided care ≥ 7 h per week	Grandparents reported being aware of generational differences in parenting practices. They disagreed with what they perceived to be a laxer and more permissive attitude toward child feeding by parents Contradicting this, many grandparents were child-centred in their approach to feeding their grandchildren. They considered children’s desires and food preferences, and some grandparents came across as permissive in their food provisioning practices Grandparents enjoyed and valued their dietary care responsibilities, feeling that they were contributing to the lives of their grandchildren. However, they believed parents had ultimate responsibility over feeding Compliance with parents’ feeding rules and practices was an important aspect of the care relationship to maintain family harmony, reduce conflict, and ensure ongoing access to grandchildren It was common for grandparents to provide their grandchildren with treats that were primarily unhealthy foods. They justified this by noting that they were indulging children’s desires and requests, rewarding good behaviour, demonstrating love, and only providing part time care (thus the treats had limited impact on the child’s nutritional health). For some grandparents, giving treats was an implicit grandparent − child social contract
Shan et al. [21]	Ireland	Quantify adults’ treat giving understanding and behaviour and compare the treat provision practices of parents, grandparents, and education practitioners	Quantitative Researcher-administered survey of parents, grandparents, child minders, and education practitioners	*N* = 1,039 participants (61% female; 20% grandparents) with child rearing responsibilities	Among grandparents, 65% reported engaging in structured treat provision, with 59% providing treats each week and 21% providing treats every day Treat foods were most commonly provided by grandparents when grandchildren asked for them (47%) and to reward good behaviour (44%). Grandparents were more likely than parents to report using treat foods to show love and care (34% vs. 22%) The most common treat foods provided by grandparents were chocolates (42%), ice-cream/ice-lollies (39%), sweets (37%), and biscuits (32%)

^a^Only findings relating specifically to the role of grandparents in children’s dietary health are reported in this table

### Provision of Meals and Snacks

Grandparents were found to frequently provide their grandchildren with meals and snacks [18•, 19]. For example, a study by Jongenelis et al. [18•] found that 98% of surveyed grandparents reported ‘usually’ providing at least 1 meal or snack to the grandchildren for whom they provide care. Snack provision was most common (82%), followed by lunch provision (57%) and then dinner (48%). McArthur et al. [19] examined the number of snacks provided by grandparents, with an average of 2.75 snacks provided by grandparents each caregiving occasion. Nearly one-fifth of grandparents reported providing 4 or more snacks per caregiving occasion.

### Types of Foods Provided

The provision of ‘treat foods’ high in sugar or fat (e.g. chocolate, sweets, ice-cream, and sugary drinks) was explored in multiple studies [20–27]. Such foods were found to play a significant role in grandparents’ food provision [22]. Indulging children with treat foods was considered by both parents and grandparents to be part of the grandparental role, with grandparents reporting that it was their right to spoil their grandchildren with such foods [24–26, 28, 29••, 30••]. Treats were found to be embedded in grandparent–grandchild routines and relationships, with many studies finding the provision of such foods to be a means through which grandparents expressed their love and care [21–23, 29••, 30••]. Nutritious meals were still prepared by grandparents for their grandchildren, but treat foods were used to strengthen the relationship bond.

Some grandparents believed it was acceptable to indulge their grandchildren as they were not primarily responsible for food provision [25, 30••, 31, 32]. They sought to counterbalance the strict rules of parents with a more lenient approach to grandchild feeding [30••]. Other motivators behind treat provision included (i) the belief that restricting treat foods created a desire for them, and that exposure provided an important means by which children learnt about moderation and self-control [29••] and (ii) rewarding good behaviour and accomplishments [22, 29••, 30••].

Not all findings suggested that grandparents’ food provision was problematic [18•, 22, 23, 29••, 32, 33]. In a study by Jongenelis et al. [18•], grandparents reported serving their grandchildren healthy foods and beverages (e.g. fresh fruit; milk, cheese, or yoghurt; vegetables; grain and cereal foods) more frequently than unhealthy foods and beverages (e.g. sugary drinks). In a study by Knight et al. [32], some children and their mothers reported consuming a greater variety of food because of grandparents’ direct involvement in providing them with meals. In other studies, some grandparents reported feeling a strong sense of responsibility to assist parents with raising healthy children and thus engaged in food provision practices they believed enhanced their grandchildren’s wellbeing [23, 29••].

#### Comparisons Between Grandparents and Parents

Mixed results were observed in studies that compared grandparents’ and parents’ food provision [22, 28, 34••]. In a study by Marr et al. [34••], there were no significant differences in the nutritional content of meals and snacks served by grandparents compared to parents. By contrast, in a study by Eli et al. [28], both grandparents and parents reported that grandparents were more likely than parents to provide children with unhealthy foods and beverages on a regular basis. In a study by O’Donohoe et al. [22], both grandparents and parents agreed that grandparents generally had more time for cooking meals from scratch whereas busy parents did not.

### Feeding Practices

Several studies explored the feeding practices of grandparents [22, 24, 25, 29••, 34••, 35••, 36–39]. Grandparents appeared to use positive feeding practices (i.e. practices that lead to favourable dietary behaviours) more often than negative feeding practices (i.e. practices that lead to unfavourable dietary behaviours). The promotion of balance and variety was the most frequently used positive feeding practice [34••, 35••]. Other positive feeding practices in which grandparents engaged included modelling healthy eating, monitoring children’s food intake, providing a healthy eating environment by making healthy foods available and limiting the amount of unhealthy foods available, teaching about nutrition, and praising children for healthy eating [22, 24, 25, 29••, 34••, 35••, 36, 37].

In terms of negative feeding practices, a study by Jongenelis et al. [35••] observed scores above the midpoint for just one negative feeding practice—control over eating. All other negative feeding practices (pressure to eat, instrumental feeding, emotional feeding) were below the midpoint. Similarly, most studies have found that using food to ameliorate negative emotions is an uncommon feeding practice among grandparents [29••, 34••, 38].

Just one study appears to have examined the association between grandparents’ feeding practices and the diet quality of their grandchildren. In this study by Jongenelis et al. [35••], positive feeding practices were identified as being more important correlates of diet quality than negative feeding practices. The provision of a healthy food environment emerged as the most important positive feeding practice; it was found to be positively associated with grandchild fruit and vegetable consumption and negatively associated with grandchild savoury and sweet snack consumption. Limit setting was also found to be important, with grandparents who engaged in this feeding practice reporting that their grandchild consumed fewer savoury snacks and sugary drinks. Mixed results were observed for other feeding practices.

#### Comparisons Between Grandparents and Parents

Some studies compared the feeding practices of grandparents and parents [34••, 36, 38]. In terms of positive feeding practices, findings suggested that grandparents were significantly more likely than parents to report creating a healthy eating environment [34••, 36]. They were also more likely to allow children to have control during mealtimes [36]. However, grandparents were less likely to encourage balance and variety, model healthy eating, monitor child food intake, and teach about nutrition [34••, 36, 38]. In terms of negative feeding practices, grandparents were less likely than parents to report using food as a reward [36]. However, they were more likely than parents to report (i) using food to regulate emotions and (ii) restricting food due to weight concerns [36, 38].

### Feeding Style

Just one study examining the feeding styles of grandparent care providers was found. In this study by Marr et al. [34••], the most common feeding style reported by grandparents was ‘indulgent’ (41%), followed by authoritative (23%), then uninvolved and authoritarian (both 18%).

### Family Disagreement over Food Provision

An important finding that was identified in many studies was the differing opinions regarding child feeding held by parents and grandparents and the potential for this to (i) prevent the adoption of healthy dietary practices and/or (ii) result in the adoption of unhealthy dietary practices [23, 25, 28, 31, 39, 40]. While grandparents generally believed that parents had ultimate authority over feeding and reported respecting the decisions of parents regarding their grandchildren’s food options [22, 24, 25, 28, 29••, 30••, 41•], the extent to which they complied with parents’ feeding instructions varied. For example, in a study by Bektas et al. [31], most grandmothers reported disagreeing at times with parents’ instructions for what and how to feed their child. They thus ignored these instructions, providing their grandchildren with food and drinks that parents did not allow (e.g. sweets, processed foods, and fruit juice), usually in secret. In other studies, grandparents reported engaging in only “minor subversions” of parents’ feeding rules [22, 28].

In parents’ reports of grandparent feeding, it was noted that grandparents held permissive attitudes towards their grandchildren’s eating habits [40]. This reportedly resulted in (i) restrictions of certain foods being inconsistently enforced and (ii) parents’ efforts to promote healthy eating habits being contradicted [40, 42]. The indulgent behaviours of grandparents were seen as problematic, a barrier to healthy eating, and a source of conflict and frustration [26, 32, 41•]. While parents noted that they had the final say, this did not come easy [41•]. Parents also noted that conflicting beliefs regarding food provision put pressure on them to adopt undesirable feeding behaviours [41•]. Mothers from culturally and linguistically diverse groups additionally reported struggling with their children’s grandparents when they fed children non-traditional foods [42].

Both grandparents and parents were reluctant to discuss their differences openly, fearing family conflict [31]. Parents were reliant on grandparents for childcare and did not want to impose on grandparent–grandchild relationships [32]. Grandparents wished to maintain family harmony and ensure ongoing access to their grandchildren [29••, 30••]. Accordingly, both parties reported complying with the other even when they did not agree [29••, 30••].

## Conclusions

Findings from research published over the last decade suggest that grandparents are exerting significant influence on child dietary health. They frequently provide their grandchildren with meals and snacks and engage in many of the same feeding practices used by parents. Although grandparents report providing their grandchildren with healthy foods, the provision of treat foods high in sugar or fat was a common finding across multiple studies. This provision led to family conflict, with the indulgent behaviours of grandparents seen by parents as a barrier to healthy eating and a source of frustration.

As grandparents become increasingly important providers of childcare globally, efforts are needed to ensure they are considered key stakeholders in the promotion of healthy eating and are targeted in policies and programs addressing children’s diets. While grandparents may not perceive themselves to be primarily responsible for child feeding, the high volume of care in which they engage means the frequent provision of treat foods could be problematic. It is promising, however, that grandparents appear motivated to assist parents with raising healthy children. Communicating to grandparents their importance and encouraging them to become champions of healthy eating may be a means by which motivation can be increased.

Research that determines how to best support grandparents to foster healthy lifestyle behaviours in children would make an important contribution to efforts to prevent of poor diet and improve health outcomes. The development of intergenerational programs that recognise the influence of all caregivers and encourage them to contribute to the goal of promoting healthy eating in children is also worthy of consideration. Such programs can assist with identifying caregiver differences that may be undermining efforts to provide children with a healthy food environment. They can also be used to optimise communication between caregivers and thus represent a potential means by which (i) the intergenerational conflict that serves as a barrier to promoting healthy eating in children may be reduced and (ii) the likelihood of children receiving congruent messages from all family members involved in their care can be increased.

It must be noted that this review explored studies published in English and only included work on non-residing grandparents. Accordingly, population groups in which co-residence of grandparents is common (e.g. South-East Asian, Chinese Asian, and South and Central Asian groups) were not represented. The influence of grandparents on grandchildren’s dietary health is likely to differ among these groups [43] and the conclusions drawn here cannot be generalised.

To conclude, the clear contribution of grandparents to children’s dietary health highlights the importance of including these caregivers in family interventions addressing lifestyle behaviours. Efforts are urgently needed to develop appropriate and effective tools that increase grandparents’ engagement in practices that support children to adopt positive behaviours and live healthy lives.

## Supplementary Information

Below is the link to the electronic supplementary material.

Supplementary file1 (DOCX 17 kb)
