# Supplementary material for: The Influence of Grandparents on Children’s Dietary Health: A Narrative Review
Source: Curr Nutr Rep. 2023 Jun 17;12(3):395–406. doi: 10.1007/s13668-023-00483-y (PMC10444634; doi:10.1007/s13668-023-00483-y)
Supplement: Supplementary file 1 — Supplementary file1 (DOCX 17 kb) [file 13668_2023_483_MOESM1_ESM.docx]

**Supplementary Material**

Aubel, J., Martin, S. L., & Cunningham, K. (2021). Introduction: a family systems approach to promote maternal, child and adolescent nutrition. *Maternal & Child Nutrition*, *17*(S1). doi: 10.1111/mcn.13228. **This review explored research that employed an ecological, family systems approach to either (i) investigate maternal, child, or adolescent nutrition or (ii) design interventions that engaged various actors within family settings.**

dos Santos, T. D., da Silva, M. O., Duarte, M. L., dos Santos, L. R., & Oliveira, A. S. B. (2022). The influence of grandparents on grandchildren. Scoping review. *Revista Neurociências*, *30*, 1-30. doi: 10.34024/rnc.2022.v30.13515. **This scoping review identified, evaluated, and summarised cross-sectional research exploring the influence of grandparents on grandchildren.**

Freeland-Graves, J., Jacobvitz, D. B., & Sachdeva, P. (2018). Role of grandparents in childhood obesity during first two years of life. *Journal of Nutrition and Health Food Science*, *6*, 1-11. doi: 10.15226/jnhfs.2018.001141. **This review explored the influence of grandparents on the weight status of grandchildren aged 0-2 years. The authors synthesised findings relating to several areas of influence, notably the effects of grandparents’ support about nutrition, cooperative co-parenting, knowledge, attitudes, beliefs, and body mass index on feeding practices and weight gain.**

Marr, C., Reale, S., Breeze, P., & Caton, S. J. (2021). Grandparental dietary provision, feeding practices and feeding styles when caring for preschool‐aged grandchildren: a systematic mixed methods review. *Obesity Reviews*, *22*(4). doi: 10.1111/obr.13157. **This systematic, mixed methods review explored grandparental dietary provision, feeding practices, and feeding styles when caring for their pre-school aged grandchildren.**

Nieri, T., Zimmer, A., Vaca, J. M., Tovar, A., & Cheney, A. (2022). A systematic review of research on non-maternal caregivers’ feeding of children 0-3 years. *International Journal of Environmental Research and Public Health*, *19*(21). doi: 10.3390/ijerph192114463. **This systematic review explored non-maternal caregivers’ feeding of children aged 0-3 years and identified issues specific to non-maternal caregivers that are unlikely to be addressed by interventions developed for mothers.**

Novak, J. R., Pratt, K., Hernandez, D. C., & Berge, J. M. (2022). Family systems and obesity: a review of key concepts and influences within and between family subsystems and a call for family‐informed interventions. *Journal of Family Theory & Review*. doi: 10.1111/jftr.12474. **This review explored influences within and between family subsystems, integrating findings into a Family Systems Theory framework. The authors presented recommendations for future work intended to target weight and weight-related behaviour in parents, children, and families.**

Pulgaron, E. R., Marchante, A. N., Agosto, Y., Lebron, C. N., & Delamater, A. M. (2016). Grandparent involvement and children’s health outcomes: The current state of the literature. *Families, Systems, & Health*, *34*(3), 260-269. doi: 10.1037/fsh0000212. **This review gathered and synthesised research on the effects of grandparent involvement on children’s physical health outcomes.**

Young, K. G., Duncanson, K., & Burrows, T. (2018). Influence of grandparents on the dietary intake of their 2–12‐year‐old grandchildren: A systematic review. *Nutrition & Dietetics*, *75*(3), 291-306. doi: 10.1111/1747-0080.12411 **This systematic review explored the influence of grandparents on the dietary intake of their grandchildren aged 2 to 12 years. The authors concluded that grandparents in caregiving roles may negatively influence grandchildren’s dietary intake and weight status.**
